# Multimodal approach to the diagnosis of pure neural neuropathy in leprosy

**DOI:** 10.3389/fmed.2025.1645960

**Published:** 2025-08-06

**Authors:** Clarissa Neves Spitz, Izabela Jardim Rodrigues Pitta, Isadora Versiani, Anna Maria Sales, Ana Caroline Siquara de Sousa, Mariana Andrea Hacker, Euzenir Nunes Sarno, Roberta Olmo Pinheiro, Marcia Rodrigues Jardim

**Affiliations:** ^1^Leprosy Laboratory, Oswaldo Cruz Institute, Oswaldo Cruz Foundation, Rio de Janeiro, Brazil; ^2^Post-Graduate Program in Neurology, Federal University of the State of Rio de Janeiro, Rio de Janeiro, Brazil; ^3^Pedro Ernesto University Hospital, Rio de Janeiro State University, Rio de Janeiro, Brazil; ^4^Department of Neurology, Antonio Pedro University Hospital, Fluminense Federal University, Niterói, Brazil; ^5^National Institute of Science and Technology on Neuroimmunomodulation, Oswaldo Cruz Institute, Oswaldo Cruz Foundation, Rio de Janeiro, Brazil

**Keywords:** leprosy, pure neural form, diagnostic tools, histopathology, neurophysiology, ultrasound

## Abstract

**Introduction:**

Neuropathy is an important feature of leprosy, a chronic infection caused by *Mycobacterium leprae* that mainly affects the skin and peripheral nerves. There is a rare and atypical form called the pure neural form, which is manifested only by changes in the nerves, without lesions on the skin, which makes early diagnosis difficult. If left untreated, neuropathy can lead to deformities and disabilities.

**Methods:**

Nerve conduction studies, ultrasound and histopathology by nerve biopsy (gold standard).

**Results:**

This study evaluated 29 cases that underwent peripheral nerve biopsy, which were then divided into positive or negative groups for pure neural form. The analysis sought to identify patterns that would help differentiate this form of leprosy, through clinical, electrophysiological and ultrasound evaluations. Of the 29 patients included, 13 were diagnosed with pure neural form and 16 were not.

**Discussion:**

The clinical features: sensory involvement occurred in all cases of confirmed pure neural form, while weakness was noted in the majority of cases in both groups, neural thickening was seen more frequently in the positive cases. Neurophysiology: sensory involvement was observed in all cases, motor involvement, a similar distribution was observed between the two groups (patterns were analyzed). On Ultrasound, neural thickening was observed in both groups, hypoechogenicity and heterogeneous fascicular disarray more frequent in the group with the diagnosis, flow in the Power Doppler in two patients only in pure neural form positive. There were no statistically significant differences in the clinical-electrophysiological and ultrasound analysis. With regard to the histopathological findings, bacilliferous findings were observed in 11 and inflammatory findings in 2. The characteristics of the negative cases were also analyzed. Pure neural leprosy remains a diagnostic challenge, especially in areas with limited resources. Histopathological examination remains the gold standard and, although non-invasive methods are desirable, they require rigorous validation to ensure accuracy.

## Introduction

Neuropathy in leprosy is the main feature of the disease. Leprosy is a chronic infection caused by *Mycobacterium leprae*, which mainly affects the skin and peripheral nerves.

The pure neural form of leprosy is a variant in which the disease manifests itself exclusively through changes in the nerves, without obvious involvement of the skin, which can make early diagnosis difficult. Neuropathy can lead to deformities and disabilities if not treated properly, due to the progressive damage to the peripheral nerves. PNL is an atypical and rare form of the disease, so its diagnosis and treatment are challenging. In terms of prognosis, early and appropriate treatment can result in a significant improvement in symptoms and the prevention of serious sequelae (1–3). Therefore, early diagnosis and continuous monitoring are crucial to avoid long-term complications (2). In cases of PNL, the cardinal finding of leprosy, neuritis, is rare in PNL (4), which makes the suspicion of the disease even lower. The clinical presentation of neuropathy can range from a single mononeuropathy to extensive nerve damage involving multiple nerves. Thus, the differential diagnosis of other neurological conditions can be challenging.

Nerve conduction studies (NCS) are essential for determining the extent of neuropathy and assessing the degree of nerve damage in leprosy (2). Demyelinating patterns are reported to be more common at the onset of leprosy neuropathy, but the presence of axonal loss is widely described in the literature as a sign of chronic neuropathy related to the disease.

Technological advances in ultrasound (US) diagnosis and high-frequency transducers now allow peripheral nerves to be traced along their entire length with structural delineation down to the fascicular level. In addition, the use of ultrasound can allow the detection of nerve thickening before it can be palpated clinically and without subjective difficulties. Morphological features such as echogenicity, fascicular pattern, cross-sectional area (CSA), Power Doppler evaluation, the appearance of the epineurium, as well as its anatomical relationships and the analysis of selected muscles are features to be investigated on ultrasound in suspected or confirmed cases of leprosy (5, 6).

Currently, nerve biopsy is the gold standard for diagnosing PNL. Its sensitivity can be increased by the PCR test. The presence of BAAR in the biopsied nerve is one of the hallmarks of leprosy histopathology and makes the diagnosis of PNL almost unequivocal, but they are only present in a small percentage of PNL patients (7). Nerve biopsy has an increased chance of diagnosis when the results are interpreted in the context of relevant clinical, epidemiological, electroneuromyographic and laboratory data [i.e., *M. leprae* DNA determined with (PCR) (2)].

The objective of this study was to correlate clinical neurological findings, nerve conduction studies and peripheral nerve ultrasound results with peripheral nerve biopsy findings (the gold standard) to establish their role in the diagnosis of the pure neural form of leprosy.

## Methodology

### Ethics statement

The study was approved by the Research Ethics Committee of the Oswaldo Cruz Institute (52641821.4.3001.5259). All patients voluntarily provided their written informed consent.

### Patient selection

At the Souza Araújo Outpatient Clinic (ASA), Rio de Janeiro, Brazil, a leprosy referral center, 450 patients underwent neurological evaluation over an 18-month period (June/2022 to December/2023). All patients were initially screened by the dermatology team. After extensive differential diagnosis, those with clinical signs suggestive of peripheral nerve involvement, without skin lesions, associated with nerve conduction studies showing specific sensory or sensorimotor impairment were included. Cases with complaints not compatible with peripheral neuropathy and those whose nerve conduction studies were normal (suggesting small fiber neuropathy) were excluded—these cases were followed up regularly at the neurology outpatient clinic.

Twenty-nine selected cases were referred for peripheral nerve biopsy (the gold standard).

The patients selected followed the flow of care at the health unit and the leprosy investigation protocols described below.

1. Clinical history.

A neurological consultation was carried out and an anamnesis was taken. Patients were asked about the time of onset of symptoms, the occurrence of neural pain and triggering factors, the presence of sensory and/or motor symptoms.

2. Clinical neurological examination.

A clinical neurological examination was carried out, with special emphasis on examining the peripheral nerves. The palmar and plantar surfaces were assessed for the presence of cyanosis or erythroderma. Tactile sensitivity, tendon reflexes, vibration sensitivity, and strength were tested according to the protocol described by Vital et al. (8). The DN4 (Douleur Neuropathique 4 Questionnaire) scale validated for Portuguese was applied (9). The assessment of complaints such as spontaneous pain or paresthesia was recorded using an 11-point Likert scale (zero = no symptoms, 10 = worst sensation imaginable).

3. Nerve conduction studies.

They were then sent for NCS of the four segments. The examinations were carried out on the same Nihon-Kohden-Neuropack S1 four-channel apparatus and the room temperature remained between 20 and 30°C. The aim of the complementary examination was to identify and characterize the lesion (sensory and/or motor) as well as the pattern of involvement. The findings of the nerve conduction study were classified according to Vital et al. (8).

4. Imaging examination.

Neuromuscular US scans were carried out using a Saevo 5 device with a 7–14 MHz linear transducer and Power Doppler parameters with gain adjustment. All examinations were carried out by the same operating physician. The peripheral nerves (indicated on the NCS) were assessed bilaterally and throughout the limb, in transverse and longitudinal sections. The cross-sectional area of the nerve, measured in square millimeters, was measured at anatomical points and at the sites of greatest thickening, and was obtained by freehand delimitation of the internal echogenic edge of the nerve. The measurements were evaluated according to the reference values (10). The fascicular pattern of the nerve and the echogenicity of the epineurium were also analyzed. The normal pattern was characterized by the presence of hypoechoic fascicles surrounded by hyperechoic tissue. The altered nerve showed hypoechoic or hyperechoic foci, focal thickening or loss of the fascicular pattern, and, eventually, hyperechogenicity and/or thickening of the epineurium. Power Doppler was used to assess the occurrence of intrafascicular and perineural vascular flow. Finally, the muscles innervated by the corresponding nerve were assessed and their morphological characteristics, echogenicity and volume were analyzed.

5. Histopathology.

The diagnosis of PNL was made by peripheral nerve biopsy. The diagnosis was made based on the criteria of Antunes et al. (3). The identification of acid-fast bacilli (AFB) is done using Wade’s special stain, Ziehl–Neelsen or Fite-Faraco, with preference for the Fite-Faraco method. It can be intact or fragmented (granular) and is quantified using the bacilloscopic index (BI), which counts the number of bacilli in a given number of fields (BI = 0 to 6+). For the diagnosis of leprosy in nerve biopsies, the criteria of Antunes et al. (3) were considered, which describes five possible histopathological categories: (1) confirmed diagnosis: presence of an inflammatory infiltrate composed of macrophages with AFB positive (+) bacilli or Schwann cells, distributed throughout the layers of the nerve; (2) highly probable diagnosis: presence of epithelioid granulomas, but AFB negative (−); (3) probable diagnosis: presence of inflammatory infiltrate composed exclusively of mononuclear cells without transformation into epithelioid cells or AFB + macrophages in the layers of the nerve, particularly in the endoneurium; (4) possible diagnosis: non-inflammatory histopathological changes were the only findings on examination of the sample (reactive morphological changes); (5) nerves with normal histological appearance.

As a complementary analysis, molecular biology was carried out in the form of PCR (polymerase chain reaction) on all the biopsied peripheral nerve samples. This analysis is not considered a diagnostic criterion, but was carried out in order to be another research tool, as described by Martinez et al. (11) and Jardim et al. (12).

After carrying out complementary tests, the patients were divided into two groups: 13 positive for the pure neural form of leprae and 16 negative.

Both groups were analyzed according to their clinical, electrophysiological, and ultrasound descriptions in order to identify possible patterns that could differentiate them.

### Statistical analysis

Double entry tables were created to cross categorical variables in order to investigate the association between the pure neural form and motor and neurological symptoms and ultrasound alterations. Fisher’s exact test and the chi-square test were used to verify statistical significance. For the continuous variables, means and standard deviations were calculated and the Mann–Whitney test was used. The significance level adopted was 5%.

## Results

### Clinic

It was observed that the mean age in PNL positive was 53 years and in PNL negative 60 years, with the majority in both groups being male (84.6 and 75%, respectively). Bacilloscopy showed an index negative (zero) in both groups. The duration of neuropathy symptoms varied between the 2 groups, being 55 months in positive PNL and 25 in negative ones, as described in Table 1.

**Table 1:** Distribution of groups.

Epidemiological data	PNL positive (*n* = 13)	PNL negative (*n* = 16)
Median age (years)	53 (37–75)	60 (33–83)
Gender	Male 11 (84.6%)	Male 12 (75.0%)
Bacilloscopy index	0	0
Symptom time (months)	55	25

PNL, pure neural leprae form.

Regarding clinical symptoms and clinical examination, the presence of neural pain was present in 61.5% of the PNL cases, while in the negative cases, 81.2% were. No case presented pain with nociceptive characteristics, suggesting neuritis. The most frequent complaint was spontaneous pain, all with a visual analogue scale (VAS) > 6; no descriptive pattern was observed for pain, with stabbing, shooting, burning, and shock reported by patients in both groups.

When asked about sensory complaints, 24 of the 29 selected patients complained of paresthesia. Sensory impairment and muscle weakness (in the respective neurologic area or the presence of atrophy) were also analyzed. A high prevalence of sensory and motor involvement was observed in both groups, with sensory involvement being unanimous in those with confirmed PNL. Weaknesses were found in the majority of cases in both groups (53.8% in PNL positive group and 62.5% in the negative group). Although neural thickening was seen more frequently in PNL positive totaling 53.8%, compared to the other group 31.2%, the data were not statistically significant. These data are detailed in Table 2.

**Table 2:** Comparative clinical characteristics between groups with and without PNL.

Clinical features	PNL positive (*n* = 13)	PNL negative (*n* = 16)	*p*-value
Pain	5 (38.5%)	3 (18.8%)	0.406
Sensitive impairment	13 (100%)	14 (87.5%)	0.488
Motor impairment	7 (53.8%)	10 (62.5%)	0.638
Neural thickening	7 (53.8%)	5 (31.2%)	0.219

PNL, pure neural leprae form.

### Neurophysiology

The patients were then referred for NCS to evaluate the affected nerve which underwent biopsy in the future. Of the 29 cases, sensory involvement was observed in all, and it was not possible to calculate the *p*-value because it is a constant. The findings either form a reduction in the amplitude of SNAPs or an absence of response (18 patients). Regarding motor impairment, a similar distribution was observed between the two groups, with the most frequent pattern being mixed (axonal and demyelinating): 53.8% in PNL positive cases and 62.5% in the negative. In cases where the motor response pattern was predominantly demyelinating, it was noted that all presented conduction block (being two cases in the positive PNL group and three in the negative PNL group). These findings are shown in Table 3.

**Table 3:** Comparative NCS between groups with and without PNL.

NCS	PNL positive (*n* = 13)	PNL negative (*n* = 16)	*p*-value
SNAPs	13 (100%)	16 (100%)	*
CMAPs	10 (76.9%)	15 (93.8%)	0.299
*Injury pattern (A)	4 (30.8%)	3 (18.8%)	0.753
*Injury pattern (D)	2 (15.4%)	3 (18.8)	
*Injury pattern (M)	7 (53.8%)	10 (62.5%)	

PNL, pure neural leprae form; SNAPs: sensory action potentials, CMAPs: compound muscle action potential; injury pattern A: axonal, D: demyelinating, M, mixed; *p*-value from Fisher’s exact test and Pearson chi-square. *No statistics are computed because it is constant.

### Ultrasound

In both groups, homogeneous neural thickening was observed (PNL positive: 76.9% × PNL negative: 75%); hypoechogenicity was observed in greater numbers in those who were diagnosed with PNL (61.5%); regarding the fascicular arrangement pattern, it was noted that the group without a diagnosis of pure neural thickening presented homogeneous morphology (75%) of the neural fascicles, in contrast to the group with the diagnosis, where heterogeneous fascicular disarray was seen (61.5%). These data did not show statistically significant values.

Muscle involvement, for suggestive evaluation of denervation, was not a frequent finding, being seen in only one case per group. Finally, the presence of peri/epineural flow on Power Doppler was seen in two patients whose final diagnosis was PNL; in the other group, this finding was not seen in any. These data are detailed in Table 4.

**Table 4:** Comparative ultrasound features between both groups.

US	PNL positive (*n* = 13)	PNL negative (*n* = 16)	*p*-value
Neural thickening	10 (76.9%)	12 (75%)	1.000
Hypoechogenicity	8 (61.5%)	5 (31.2%)	0.103
Fascicular pattern Hom	8 (38.5%)	12 (75%)	0.688
Fascicular pattern Het	5 (61.5%)	4 (25%)	
Flow on PD	2 (15.4%)	0 (0%)	0.192
Muscle involvement	1 (7.7%)	1 (6.2%)	1.000

PNL, pure neural leprae form; Hom, homogeneous; Het, heterogeneous; PD, power Doppler; *p*-value from Fisher’s exact.

### Histopathology

Of the 13 confirmed cases, 11 were seen with AFB and two had inflammatory findings in epineurium in histopathology. In this patient profile, the most common histopathological findings were inflammatory infiltrate with macrophages or AFB + Schwann cells (69.2%), fibrosis in all cases (most of which were concentrated in the severe grade), perineural edema (76.9%) and decrease or absence of myelin fibers (with destruction between grades). These results are listed in Table 5.

**Table 5:** Histopathological findings between groups.

Histopathology	PNL positive (*n* = 13)	PNL negative (*n* = 16)	*p*-value
(1) Inflammatory infiltrate with macrophages or AFB + Schwann cells (any layer)	9 (69.2%)	0 (0%)	0.001
(2) Epithelioid granulomas but AFB−	4 (30.7%)	0 (0%)	0.035
(3) Mononuclear inflammatory infiltrate without transformation, mainly in the epineurium or endoneurium	2 (15.4%)	4 (25%)	0.572
(4) Fibrosis (mainly in the endoneurium)	13 (100%)	15 (93.7%)	
Discreet	0 (0%)	1 (6.6%)	0.007
Mild	2 (15.4%)	7 (46.6%)	
Moderate	2 (15.4%)	6 (40%)	
Severe	9 (69.2%)	1 (6.6%)	
(5) Subperineural edema	6 (46.1%)	10 (62.5%)	0.411
(6) Perineural thickening	10 (76.9%)	6 (37.5%)	0.064
(7) Microfasciculation	5 (38.4%)	3 (18.7%)	0.277
(8) Decrease or absence of myelin fibers (semi-fine cuts)	13 (100%)	16 (100%)	
Mild	0 (0%)	2 (12.5%)	0,005
Moderate	2 (15.4%)	10 (62.5%)	
Severe	6 (46.1%)	4 (25%)	
Absence	5 (38.4%)	0 (0%)	
AFB+	11 (84.6%)	0 (0%)	0,001
PCR technique	7 (53.8%)	0 (0%)	0,001

PNL, pure neural leprae form; PCR, polymerase chain reaction; AFB, acid-fast bacilli *p*-value from Fisher’s exact test.

Cases not classified as PNL presented findings such as fibrosis, which were predominantly mild to moderate. A higher frequency of subperineural edema (62.5%) was also observed in this group. Although, as in the positive PNL cases, in which absence or reduction of myelinated fibers was observed, the distribution in the negative cases was more evenly distributed between the grades, with the moderate grade being where the largest number was concentrated. These findings are detailed in Table 5.

When complemented by the evaluation using the biomolecular PCR technique, seven of the 13 were positive, in contrast to the cases in PNL negative in which no case was positive using the method. Such data are illustrated in Table 5.

In summary as shown in Table 5, inflammatory infiltrate with macrophages or AFB + Schwann cells, epithelioid granulomas but AFB−, fibrosis, and decrease or absence of myelin fibers demonstrated statistically significant differences. The presence of acid-fast bacilli and PCR also showed statistically significant differences with *p* < 0.05.

**Figure 1:**
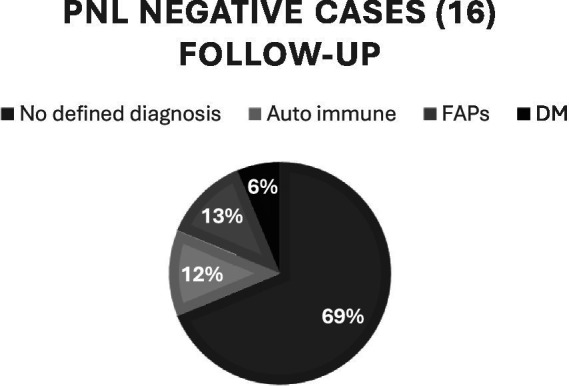
Distribution of non-PNL cases in post-biopsy follow-up. PNL, pure neural leprae form; PCR, polymerase chain reaction; FAPs, familial amyloid polyneuropathies; DM, diabetes mellitus.

Most of them, 11, have not yet had a final diagnosis, and were then referred to the originating service to continue diagnostic investigation. The others had final neuropathy secondary to diabetes mellitus, amyloidosis and autoimmune disease, as shown in the distribution in Figure 1.

## Discussion

In this study, a higher rate of suspected and confirmed cases was observed among male patients, aged between 50 and 60 years. These data are in agreement with the literature (13, 14), where from 2017 to 2021, 55.7% of the total cases occurred in males, as well as a predominance with greater frequency in individuals between 50 and 59 years old (14). This fact is generally due to greater exposure to the bacillus and a lower tendency for men to seek health services.

There was also a variation in the time of occurrence of signs/symptoms, with a long period between the onset of the neurological condition and investigation by a specialist, and in the group of patients with a confirmed diagnosis of leprosy, this time was more than double. Patients with the pure neural form of leprosy generally have an indolent evolution, without neuritis (cardinal sign of leprosy), which delays the search for a diagnosis and makes it difficult for doctors to suspect the disease.

Neural pain findings were similar; complaints of pain were not frequent or significant between the two groups. It is known that in leprosy, the prevalence of neural pain varies from 17 to 70.3% (15, 16). A study by Giesel et al. (14) showed an annual prevalence of 15%. Once again, the absence of neuritis may justify these findings, in addition the chronic evolution delays the appearance of neuropathic pain (1).

In contrast, sensitive complaints occurred in all PNL-positive individuals and in 87.56% of those who were negative. Motor impairment was noted in more than half of the cases in both groups although there was no statistically significant difference. Neuropathy in leprosy is known for the initial impairment of sensitivity, initially thermal and painful, and later tactile sensitivity, either in the pure neural form (nerve trunks) or in the cutaneous branches (in dermatological lesions). This can be explained by the parasitism of Schwann cells of unmyelinated fibers (C and A delta fibers) in *M. leprae* (2, 17–19).

Although neural thickening is one of the hallmarks of leprosy, it is not a defining pattern, as can be seen in the distribution of cases without statistical difference. Neural thickening and/or associated neurological deficit are also common findings in patients with chronic inflammatory neuropathy and compressive neuropathies (8, 17, 20). Giesel et al. (14) identified neural thickening in 60% of patients with leprosy and neuropathic pain. Neural thickening generally does not regress after MDT, therefore, in patients with chronic neuropathy due to leprosy, the isolated assessment of neural thickening associated with pain is not a good parameter in isolation (14, 21, 22).

Regarding neurophysiology, as the clinical findings, it was found that sensory impairment was higher than the incidence of motor dysfunction, in accordance with other studies (2, 8, 23). Van Brakel et al. (24) found that sensory nerves are compromised earlier. In this study, we saw that the most frequent pattern was mixed (axonal and demyelinating), reduced amplitude of CMAPs were found as the most important electrodiagnostic finding in leprosy neuropathy (20, 24). The chronic evolution of neuropathy in leprosy justifies findings of axonal injury; neuropathy usually occurs during episodes of acute or silent neuritis. These nerve patterns observed in the NCS highlight the complex nature of nerve damage in leprosy. The high sensitivity of NCS was also reported by other authors being a useful tool to detect and evaluate the extent of leprosy neuropathy (24, 25).

The characteristics found in the groups in relation to the ultrasound images, revealed that the neural thickening was greater than perceived in the clinical examination. This is justified by the objectivity of the method in delimiting the cross-sectional area of the nerve, without the subjective question of the examiner. Nerve thickening as an isolated finding is not pathognomonic for leprosy neuropathy; therefore, it is essential that there is a set of other associated ultrasound alterations to make this diagnosis more likely (26–28). Hypoechoic areas associated with the loss of the fascicular pattern of the nerve are other findings that can be observed in leprosy-related neuropathy (6, 28, 29). Martinoli et al. (30) found thickening of the ulnar nerve associated with fascicular abnormalities in 52% of the nerves and changes in fascicular architecture were reported as one of the most relevant findings; however, it is not known whether this is an irreversible finding after nerve injury. However, it is not a finding exclusive to leprosy neuropathy, being found in 25% of cases of neuropathies of other etiologies.

We saw that the fascicle arrangement pattern in the diagnosed groups was heterogeneous, with the fascicles being affected unevenly within the nerve, which can be explained by different neural injury processes over the period, with untreated reactive episodes and progressive axonal loss that may reflect the severe involvement of the nervous tissue by cellular infiltrates and formation of granulomas (30).

In 15% PNL group we detected flow on Power Doppler (and there were no cases in the other group). Detection of intra/perineural flow may represent a marker of active neuritis, including silent neuritis, and is directly proportional to the increase in nerve diameter (6, 28, 29).

Finally, when we look at the histopathological differences (gold standard), the detection of the bacillus was the unequivocal finding, accounting 85% of cases and in two of them characteristic inflammatory changes were seen—which defines the diagnosis as reliable even in the absence of the bacillus. The presence of AFB in the biopsied nerve is one of the features of the histopathology of leprosy and makes the diagnosis of PNL almost unequivocal, but they are present only in a small percentage of patients with PNL (2, 31).

Nerve biopsy is the gold standard for the diagnosis of PNL and its sensitivity can be increased by the PCR test (7), with the PCR test being another way to demonstrate the presence of *M. leprae.* Interestingly, in our study, 54% (PNL positive) were positive, while none were positive in negative cases. As described in other studies such as Jardim et al. (12), mycobacterial infection in these cases was most frequently demonstrated by PCR, following the observation that 60% of cases positive for PCR and anti-PGL-I of *M. leprae* were also positive for AFB, which may indicate that these patients carry a greater number of bacteria. The general data reported previously demonstrates that in patients with PNL, PCR for *M. leprae* is more sensitive than AFB microscopy in detecting *M. leprae*, also detecting patients with low bacillary load, and may be an additional instrument to be used. According to what Santos (32) reported in 1993, patients do not always present skin lesions and the detection of the leprosy bacillus in other materials would broaden the diagnosis of leprosy and regardless of the relationship between PCR positivity and the viability of *M. leprae*, PCR is much more sensitive than microscopic examination for the direct detection of bacilli and can aggregate in histopathology.

## Conclusion

Pure neural leprosy remains a diagnostic challenge, particularly in areas lacking the personnel or structural resources needed for a thorough investigation with appropriate tools. We analyzed the clinical, electrophysiological and ultrasonographic characteristics between the groups diagnosed from peripheral nerve biopsy (pure neural form and non-pure neural form) and did not identify statistically significant patterns to differentiate them. Confirming that although we are exploring alternative diagnostic tools, histopathological examination remains the gold standard for the diagnosis of PNL. We also describe the histopathological patterns between the groups in an attempt to establish the most frequently seen findings among them, as well as in cases where the disease was not diagnosed, which pathologies were subsequently identified. There is consensus on the need for non-invasive methods to diagnose this rare form of leprosy, but more rigorous scientific studies are needed to ensure accurate identification of this form of leprosy and avoid false positive diagnoses in patients.

The study has limitations due to the sample size and the convenience sample, which could impact the lack of statistical significance and generalizability of the results. On the other hand, because it uses data from patients at a leprosy referral center, the study has high internal validity and important results for raising hypotheses to be further explored in future studies.

## Data Availability

The datasets presented in this study can be found in online repositories. The names of the repository/repositories and accession number(s) can be found in the article/supplementary material.
